# Moroccan Bee Bread Improves Biochemical and Histological Changes of the Brain, Liver, and Kidneys Induced by Titanium Dioxide Nanoparticles

**DOI:** 10.1155/2021/6632128

**Published:** 2021-06-23

**Authors:** Meryem Bakour, Nawal Hammas, Hassan Laaroussi, Driss Ousaaid, Hinde EL Fatemi, Abderrazak Aboulghazi, Najoua Soulo, Badiaa Lyoussi

**Affiliations:** ^1^Laboratory of Natural Substances, Pharmacology, Environment, Modeling, Health and Quality of Life (SNAMOPEQ), Faculty of Sciences Dhar El Mahraz, University Sidi Mohamed Ben Abdellah, Fez, Morocco; ^2^Laboratory of Biomedical and Translational Research, Faculty of Medicine and Pharmacy, University Sidi Mohamed Ben Abdellah, 30000 Fez, Morocco; ^3^Department of Pathology, University Hospital Hassan II, 30000 Fez, Morocco

## Abstract

Titanium dioxide nanoparticles (TiO_2_) were used in various fields such as food industry, cosmetics, medicine, and agriculture. Despite the many advantages of nanotechnology, the adverse effects of nanoparticles are inevitable. The present study was conducted to evaluate the protective effect of bee bread on titanium dioxide (TiO_2_) nanoparticle toxicity. Male rats were randomly divided into four groups: Group 1 received daily by gavage (10 mL/kg bw) of distilled water, Group 2 received bee bread ethanolic extract (100 mg/kg bw), Group 3 received TiO_2_ (100 mg/kg bw) and distilled water (10 mL/kg bw), and Group 4 received TiO_2_ (100 mg/kg bw) and bee bread ethanolic extract (100 mg/kg bw). All treatments were given daily by gavage during 30 days. At the end of the experiment period, blood samples were collected to analyze fasting blood glucose, lipid profile (TC, TG, LDL-C, HDL-C, and VLDL-C), liver enzymes (AST, ALT, and LDH), total protein, urea, albumin, creatinine, sodium, potassium, and chloride ions. In addition, histological examinations of the kidneys, liver, and brain were investigated. The results showed that the subacute administration of TiO_2_ alone (100 mg/kg bw) had induced hyperglycemia (309 ± 5 mg/dL) and elevation of hepatic enzyme levels, accompanied by a change in both lipid profile and renal biomarkers as well as induced congestion and dilatation in the hepatic central vein and congestion in kidney and brain tissues. However, the cotreatment with bee bread extract restored these biochemical parameters and attenuated the deleterious effects of titanium nanoparticles on brain, liver, and kidney functions which could be due to its rich content on functional molecules. The findings of this paper could make an important contribution to the field of using bee bread as a detoxifying agent against titanium dioxide nanoparticles and other xenobiotics.

## 1. Introduction

Titanium dioxide nanoparticles (TiO_2_NPs) are ultrafine particles ranging in size from 1 to 100 nanometers [1]. Due to the unique properties derived from their small sizes, TiO_2_NPs are widely used in paints, toothpastes, sunscreens, food colorants, medicines, pastry, and confectionery. Although TiO_2_NPs had many beneficial applications, it had also side effects. It is toxic when it is in powder form; it irritates the respiratory tract and could be carcinogenic and harmful to the cell structure [2]. Everyone has been exposed to nanosized TiO_2_ particles, because we inhale them with each breath and we consume them with every drink and food. However, several practical questions arise when dealing with the biological inertness. Thereby, the adverse effects of TiO_2_ nanoparticles have been shown in many studies, such as neuroinflammatory effect, brain injury [3], and pulmonary toxicity [4]. Titanium dioxide nanoparticles can damage spleen, which induces oxidative stress, apoptosis, liver injury, and heart damage.

The wide uses of nanoparticles in different aspects of life (food industry and medicine) increase chronic exposure to titanium particles. The toxicity of TiO_2_NPs can be induced by several mechanisms. These small particles could be inducing genetic toxicity through changing gene expression [5–7]. Additionally, it can be increasing the expression of *cyp35a2* and dropping down the growth and fertility functions as toxicity endpoints size-dependent [8]. In the same context, the TiO_2_NPs had the ability to increase hepatic levels of interleukin 6 (IL6), nuclear factor kappa *β*, and tumor necrosis factor alpha (TNF*α*) [9].

Another study revealed that the TiO_2_NPs can inhibit cell viability and neurite growth through the reduction of glutamine activity and NR1 expression, while it is able to stimulate the phosphate-activated glutaminase activity [10]. Moreover, TiO_2_NPs may produce oxidative stress via the production of reactive oxygen species (ROS) which counteract with DNA and generate 8-OHG leading to errors and mutation in gene replication [11, 12].

The endpoints affected by TiO_2_NPs can be outlined in three mechanisms, the ROS production which induces electron-hole pairs, nanoparticle cell attachment which caused cell wall damage and lipid peroxidation, and the attachment of TiO_2_NPs to intracellular organelles and macromolecules inducing damage of cell membranes [13]. Therefore, we must find ways to reduce the impact of nanoparticles, especially those used in the food industry.

In the last years, there has been a growing interest in the use of natural products, which are widely cited in traditional medicine, yielding knowledge to derive bioactive molecules for drug discovery [14]. Bee products (honey, royal jelly, bee wax, propolis, bee pollen, and bee bread) are among the natural products that are known for their powerful healing properties such as antioxidant, nephroprotective, anti-inflammatory, and antimicrobial effects [15–17].

Bee bread is a bee product obtained by an anaerobic fermentation process of a mixture of nutrients stored in the combs, constituted by the pollen collected by bees, honey, and digestive enzymes of bees [18]. The nutritional value of bee bread is higher than that of bee pollen, and it is more digestible. Bee bread contains all the essential amino acids, rich in vitamins such as vitamin B and vitamin K; it is a good source of minerals like K, Mg, Na, Ca, P, and Mn. Bee bread also contains fatty acids, carbohydrates, and antioxidant compounds [18–20].

Numerous studies are aimed at evaluating the biological activities of bee bread, and they showed that it can be a source of many pharmacologically active molecules [21, 22]. It was demonstrated that bee bread can act as a detoxifying agent against the toxicity of aluminum [17]; it improves glycemia in diabetics [23]. *In vitro* bee bread showed many biological properties such as antimicrobial, antioxidant, and antitumoral activities [24–26].

For all these reasons, this study was aimed at evaluating the protective effect of bee bread as a natural product against biochemical changes and tissue damages of the liver, kidneys, and brain caused by titanium dioxide nanoparticles in Wistar rats. Due to its efficiency and safety, ethanol was used as a conventional solvent from “nonnatural”/petroleum resources for the extraction of bioactive compounds from the bee bread sample [27–29].

## 2. Material and Methods

### 2.1. Titanium Nanoparticles

Titanium dioxide nanoparticles (TiO_2_) were purchased from Sigma-Aldrich, Germany. The size of nanoparticles is 21 nm. TiO_2_ dose (100 mg/Kg bw) was chosen based on the findings of the toxicological studies conducted by Vasantharaja et al. [30] and Bu et al. [31].

### 2.2. Bee Bread Sample

Bee bread sample was obtained from a professional beekeeper, produced in Imouzzer Marmoucha (33°28′12^″^N and 4°17′24^″^W) Morocco; the fresh product was stored at -20°C until use. For the animal's experiment, bee bread was extracted using ethanol (70%) by maceration for a week; the filtrate was concentrated by a rotary evaporator. Distilled water was added to obtain the concentration of 100 mg/kg bw). The dose was chosen according to Doganyigit et al. [32].

The full characterization of the bee bread sample used in this study was previously published, it is classified as multifloral, it contains 18 ± 1 g/100 g of total free sugars, 19.96 ± 0.08 g/100 g of proteins, 64.7 ± 0.4% of polyunsaturated fatty acids, and 10.9 ± 0.9 mg/100 g of tocopherols, minerals (Ca, Fe, K, Mg, Na, Zn, P, Mn), and natural antioxidants (thirteen flavonol glycoside derivative, mainly methylherbacetrin, isorhamnetin, quercetin, and kaempferol derivatives); the major compound present was isorhamnetin-*O*-hexosyl-*O*-rutinoside [33].

### 2.3. Experimental Animals

Sixteen Wistar rats (body weight 150 ± 10.20 g) were used for *in vivo* experiment. The animals had free access to food and water. All animal procedures were conducted in accordance with the internationally accepted principles for laboratory animal use and care as found in the European Community guidelines (EEC Directive of 1986; 86/609/EEC). The protocol was approved by Sidi Mohamed Ben Abdellah University, Fez, Morocco (USMBA-SNAMOPEQ 2017-03), under the responsibility of the Laboratory of Natural Substances, Pharmacology, Environment, Modeling, Health and Quality of Life,

### 2.4. Experimental Design

Rats were randomly divided into four groups: Group 1 received (10 mL/kg bw) of distilled water, Group 2 received ethanolic extract of bee bread (100 mg/kg bw), Group 3 received TiO_2_ (100 mg/kg bw) and (10 mL/kg bw) of distilled water, and Group 4 received TiO_2_ (100 mg/kg bw) and ethanolic extract of bee bread (100 mg/kg bw). The interventions were provided daily by gavage for 30 days.

It is recommended for several biochemical analyses to use heparinized plasma, because firstly plasma constituents reflect better the pathologic state than serum [34] and secondly the volume of heparinized plasma is higher by 15% to 20% than serum obtained from the same volume of blood [35]. For that, blood samples were collected from each rat on day 30 by retro-orbital plexus using capillary tubes and heparin as anticoagulant; then, the blood samples were centrifuged at 4000 rpm for 10 min at 4°C, and the plasma samples obtained were conserved at -20°C until analysis.

The kidneys, brain, and liver of each rat were removed and were immediately fixed in formalin solution (10%).

### 2.5. Biochemical Methods

After 30 days of treatment, blood samples were collected for the analysis of alanine aminotransferase (ALT) (kit number 7D56-20, Alanine/NADH method); aspartate aminotransferase (AST) (kit number 7D81-20, Aspartate/NADH method); lactate dehydrogenase (LDH) (kit number 7D69-20, Lactate/NAD method); glucose (kit no 7D66-20, hexokinase method); creatinine (kit number 7D64-20, picric acid/NaOH method); urea (kit number 7D75-30, urease/NADH method); albumin (kit number 7D53-20, bromocresol Green method); total protein (kit number 7D73-20, biuret method); total cholesterol (TC) (kit number 7D62-20, cholesterol oxidase/POD method); triglycerides (TG) (kit number 7D74-20, lipase/GK/POD method); and high-density lipoprotein cholesterol (HDL-C) (kit number 7D67-20, homogeneous test method). Low-density lipoprotein cholesterol (LDL-C) was calculated according to the following formula:

(1) $$\begin{array}{c} \text{LDL}‐\text{C}=\text{TC}-\left(\text{HDL}‐\text{C}+\text{TG}/5\right). \end{array}$$

Very low-density lipoprotein (VLDL) was calculated using the following formula:

(2) $$\begin{array}{c} \text{VLDL}=\frac{\text{Triglycerides}}{5}. \end{array}$$

All investigated parameters were measured using Architect c8000i biochemistry analyzer.

Plasma, chloride (Cl^−^), sodium (Na^+^), and potassium (K^+^) were analyzed using ion-selective potentiometry method (Architect c8000i biochemistry analyzer) (kit refs 1E49-01, LN9D28-02 and 1E48-20, respectively).

### 2.6. Histological Analysis

Histological analysis of the brain, liver, and kidney was performed using the method described by Bakour et al. [36]. The organs were fixed in the formalin solution (10%) for 24 h; then, tissue samples were dehydrated using ethanol with a series of increasing concentration; after, the organs were clarified in toluene and then embedded in paraffin. A microtome was used to cut fine sections (5-6 mm) from paraffin blocks. Hematoxylin and eosin (H&E) was used for staining the slides obtained for observation under an optical microscope (model BK 5000 Trino of the Realux brand).

### 2.7. Statistical Analysis

GraphPad Software (San Diego, CA, USA) was used for statistical analysis; data were represented as the mean ± SEM. Statistical comparisons between groups were performed with one-way ANOVA followed by Bonferroni post hoc analysis. Throughout the analysis, *p* < 0.05 was considered significant.

## 3. Results

### 3.1. Effect of Bee Bread on Enzymatic Liver Changes Induced by Titanium Dioxide Nanoparticles

**Figure 1:**
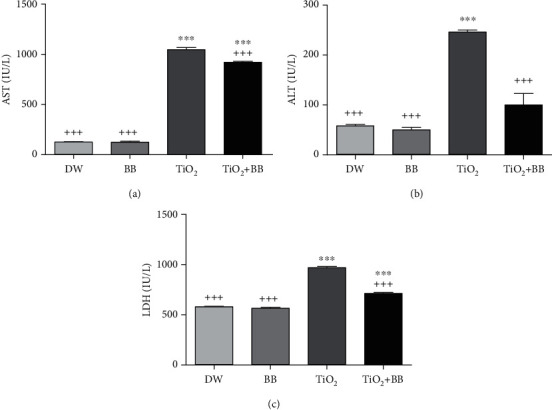
Effect of bee bread extract on liver enzymes changes induced by titanium dioxide nanoparticles. (a) AST plasma concentrations; (b) ALT plasma concentration; (c) LDH plasma concentration. DW: distilled water group; BB: bee bread group; TiO_2_: titanium dioxide nanoparticle group; TiO_2_+BB: titanium dioxide nanoparticles+bee bread group. The symbol (∗) was used to present the statistical significance of the comparison between distilled water group (DW) and all groups using one-way ANOVA followed by Bonferroni post hoc analysis (^∗^*p* < 0.05; ^∗∗^*p* < 0.01; ^∗∗∗^*p* < 0.001). The symbol (+) was used to present the statistical significance of the comparison between titanium dioxide nanoparticle group (TiO_2_) and all groups using one-way ANOVA followed by Bonferroni post hoc analysis (^+^*p* < 0.05: ^++^*p* < 0.01; ^+++^*p* < 0.001). Data are the means of three replicates and presented as the mean ± SEM.

The results of lactate dehydrogenase (LDH), alanine aminotransferase (ALT), and aspartate aminotransferase (AST) levels presented in Figure 1 revealed a significant elevation (*p* < 0.001) of these parameters in the group of rats that received TiO_2_ alone, compared with the control group. However, in the group who received the coadministration of TiO_2_ with the bee bread extract, the changes were lower compared to the group that received TiO_2_ alone, especially for ALT (Figure 1(b)).

### 3.2. Effect of Bee Bread Extract on Lipid Profile Changes Induced by Titanium Dioxide Nanoparticles

**Figure 2:**
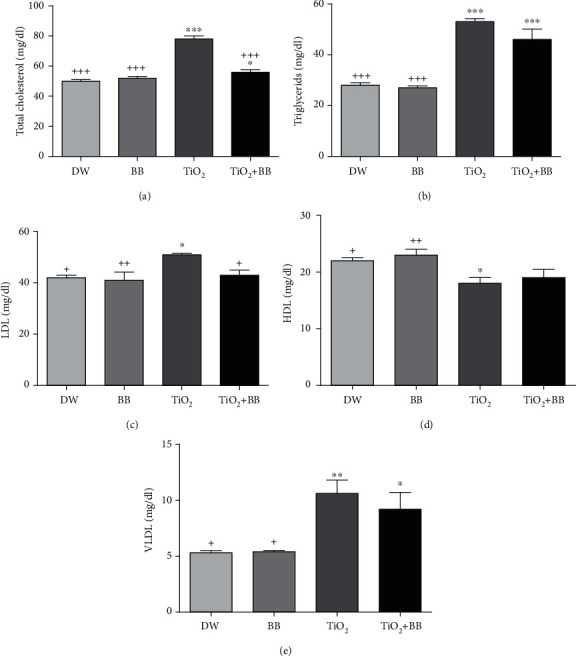
Effect of bee bread extract on lipid profile changes induced by titanium dioxide nanoparticles. (a) Total cholesterol concentration; (b) triglyceride concentration; (c) LDL concentration; (d) HDL concentration; (e) VLDL concentration. DW: distilled water group; BB: bee bread group; TiO_2_: titanium dioxide nanoparticle group; TiO_2_+BB: titanium dioxide nanoparticles+bee bread group. The symbol (∗) was used to present the statistical significance of the comparison between distilled water group (DW) and all groups using one-way ANOVA followed by Bonferroni post hoc analysis (^∗^*p* < 0.05; ^∗∗^*p* < 0.01; ^∗∗∗^*p* < 0.001). The symbol (+) was used to present the statistical significance of the comparison between titanium dioxide nanoparticle group (TiO_2_) and all groups using one-way ANOVA followed by Bonferroni post hoc analysis (^+^*p* < 0.05: ^++^*p* < 0.01; ^+++^*p* < 0.001). Data are the means of three replicates and presented as the mean ± SEM.

Figure 2 summarizes the results of lipid profile and shows a significant elevation of TC (^∗∗∗^*p* < 0.001), TG (^∗∗∗^*p* < 0.001), LDL (^∗^*p* < 0.05), and VLDL (^∗∗^*p* < 0.01) and a decrease in HDL (^∗^*p* < 0.05) levels in the group of rats that received TiO_2_ nanoparticles only, while, in the group that received simultaneously TiO_2_ nanoparticles and bee bread extract, no significant changes were recorded in TC, LDL, and HDL as compared to the control group that received distilled water, while TG and VLDL levels were changed significantly (Figures 2(b) and 2(e)).

### 3.3. Effect of Bee Bread Extract on Plasma Glucose Change Induced by Titanium Dioxide Nanoparticles

**Figure 3:**
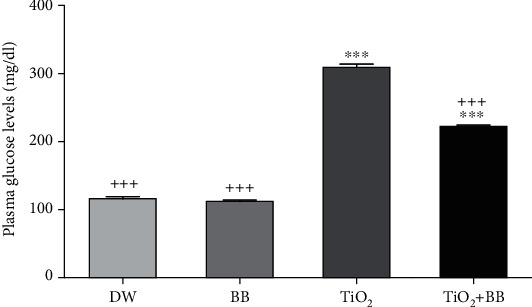
Effect of bee bread extract on plasma glucose changes induced by titanium dioxide nanoparticles. DW: distilled water group; BB: bee bread group; TiO_2_: titanium dioxide nanoparticle group; TiO_2_+BB: titanium dioxide nanoparticles+bee bread group. The symbol (∗) was used to present the statistical significance of the comparison between distilled water group (DW) and all groups using one-way ANOVA followed by Bonferroni post hoc analysis (^∗^*p* < 0.05: ^∗∗^*p* < 0.01; ^∗∗∗^*p* < 0.001). The symbol (+) was used to present the statistical significance of the comparison between titanium dioxide nanoparticle group (TiO_2_) and all groups using one-way ANOVA followed by Bonferroni post hoc analysis (^+^*p* < 0.05: ^++^*p* < 0.01; ^+++^*p* < 0.001). Data are the means of three replicates and presented as the mean ± SEM.

Figure 3 presents the levels of glycemia in the treated groups; the results revealed a significant increase (^∗∗∗^*p* < 0.001) of the plasmatic glucose level in the group received TiO_2_ nanoparticles, compared to the control group that received distilled water. However, bee bread extract combined with TiO_2_ nanoparticles reduced significantly (^+++^*p* < 0.001) the plasma glucose levels, in comparison to the group of rats that received TiO_2_ nanoparticles only.

### 3.4. Effect of Bee Bread on Plasmatic Changes of Creatinine, Urea, Total Protein, and Albumin Induced by Titanium Dioxide Nanoparticles

**Figure 4:**
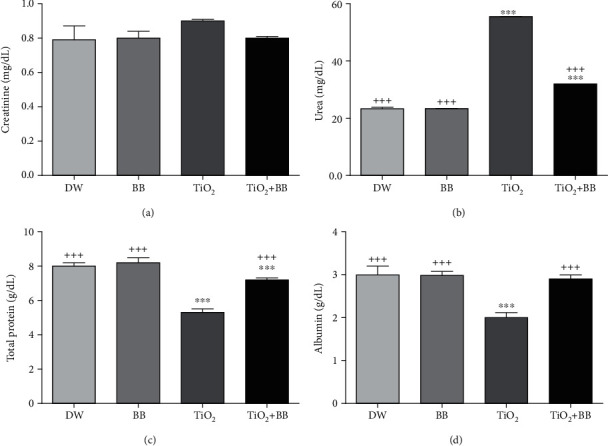
Effect of bee bread extract on plasmatic changes of (a) creatinine, (b) urea, (c) total protein, and (d) albumin induced by titanium dioxide nanoparticles. DW: distilled water group; BB: bee bread group; TiO_2_: titanium dioxide nanoparticle group; TiO_2_+BB: titanium dioxide nanoparticles+bee bread group. The symbol (∗) was used to present the statistical significance of the comparison between distilled water group (DW) and all groups using one-way ANOVA followed by Bonferroni post hoc analysis (^∗^*p* < 0.05; ^∗∗^*p* < 0.01; ^∗∗∗^*p* < 0.001). The symbol (+) was used to present the statistical significance of the comparison between titanium dioxide nanoparticle group (TiO_2_) and all groups using one-way ANOVA followed by Bonferroni post hoc analysis (^+^*p* < 0.05; ^++^*p* < 0.01; ^+++^*p* < 0.001). Data are the means of three replicates and presented as the mean ± SEM.

As shown in Figure 4, the subchronic administration of TiO_2_ caused a significant increase (^∗∗∗^*p* < 0.001) in the plasmatic urea level (Figure 4(a)) and a significant decrease in total protein (^∗∗∗^*p* < 0.001) (Figure 4(c)) and albumin levels (^∗∗∗^*p* < 0.001) (Figure 4(d)), while no significant change was observed in creatinine plasmatic levels. The group of rats that received TiO_2_ nanoparticles combined with bee bread extract revealed significant protection (^+++^*p* < 0.001) against the biochemical changes compared to the group received TiO_2_ nanoparticles only.

### 3.5. Effect of Bee Bread on Plasmatic Changes of Sodium, Potassium, and Chloride Induced by Titanium Dioxide Nanoparticles

**Figure 5:**
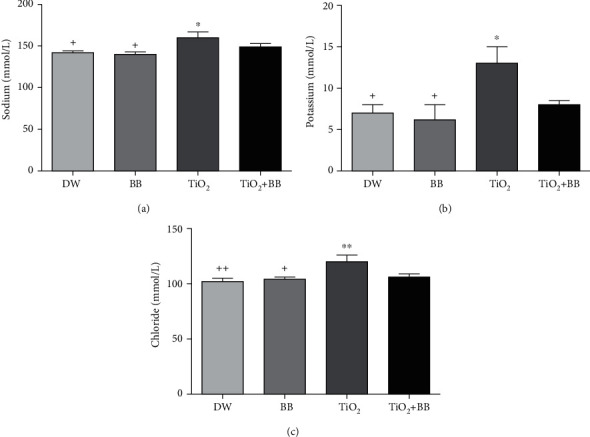
Effect of bee bread extract on plasmatic changes of (a) sodium, (b) potassium, and (c) chloride induced by titanium dioxide nanoparticles. DW: distilled water group; BB: bee bread group; TiO_2_: titanium dioxide nanoparticle group; TiO_2_+BB: titanium dioxide nanoparticles+bee bread group. The symbol (∗) was used to present the statistical significance of the comparison between distilled water group (DW) and all groups using one-way ANOVA followed by Bonferroni post hoc analysis (^∗^*p* < 0.05; ^∗∗^*p* < 0.01; ^∗∗∗^*p* < 0.001). The symbol (+) was used to present the statistical significance of the comparison between titanium dioxide nanoparticle group (TiO_2_) and all groups using one-way ANOVA followed by Bonferroni post hoc analysis (^+^*p* < 0.05: ^++^*p* < 0.01; ^+++^*p* < 0.001). Data are the means of three replicates and presented as the mean ± SEM.

Plasmatic concentrations of sodium, potassium, and chloride of the studied groups are summarized in Figure 5. The plasmatic levels of sodium, potassium, and chloride were significantly increased in the group received TiO_2_ nanoparticles only compared to the control group, while no significant increase was observed in the group coadministrated with bee bread extract combined and TiO_2_ nanoparticles.

### 3.6. Histological Analysis

**Figure 6:**
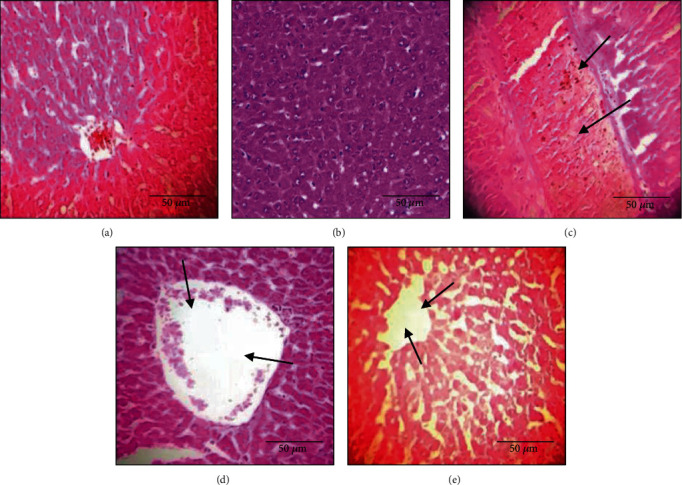
Histological evaluation of the liver. The arrows represent pathological changes in the tissue: (a) control group—×100; (b) BB group: normal tissue—×100; (c) TiO_2_ group: congestion—×100; (d) TiO_2_ group: dilatation in the central vein—×100; (e) TiO_2_+BB group: dilatation in the central vein—×100.

The histological analysis of liver tissue (Figure 6) indicated that subacute administration of TiO_2_ nanoparticles alone induced congestion (Figure 6(c)) and dilatation in the central vein (Figure 6(d)). However, this dilation was less (Figure 6(e)) in rats cotreated with bee bread extract (Group 4).

**Figure 7:**
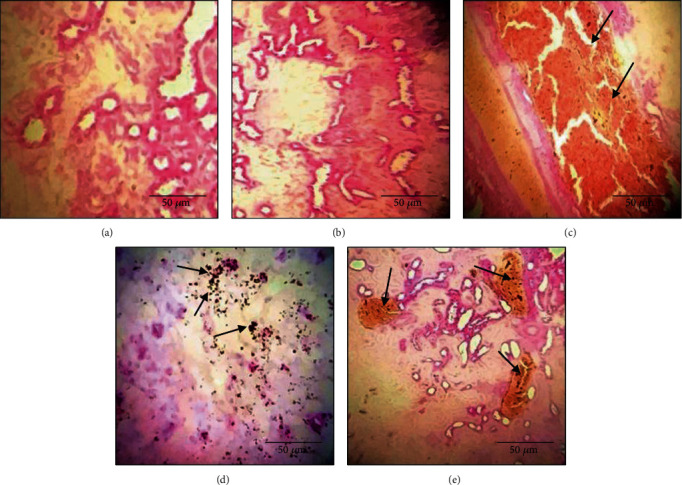
Histological evaluation of the kidney. The arrows represent pathological changes in tissue: (a) control group—×100; (b) BB group: normal tissue—×100; (c) TiO_2_ group: congestion—×100; (d) TiO_2_ group: particles of agglomerated TiO_2_—×100; (d) TiO_2_+BB group: congestion—×100.

The histological analysis of the kidney tissue (Figures 7) showed that TiO_2_ induced congestion when administered alone (Figure 7(c)). However, the coadministration of bee bread extract with TiO_2_ for 30 days has reduced the intensity of congestion in renal tissue (Figure 7(e)). In addition, as shown in (Figure 7(d)), particles of TiO_2_ appeared as agglomerates in the kidney tissue of untreated rats.

**Figure 8:**
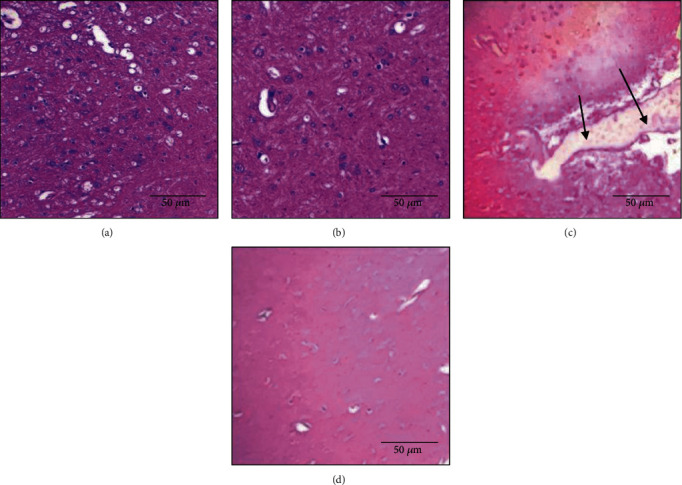
Histological evaluation of the brain. The arrows represent pathological changes in tissue: (a) control group—×200; (b) BB group: normal tissue—×200; (c) TiO_2_ group: congestion—×100; (d) TiO_2_+BB group: normal tissue—×100.

Concerning the histological analysis of brain tissue, the results presented in Figure 8 showed that titanium dioxide nanoparticles induced congestion in the brain tissue when administrated alone in rats (Figure 8(c)), while the coadministration of bee bread extract with TiO_2_ has protected the brain against this histopathological changes.

## 4. Discussion

TiO_2_ nanoparticles have been used in a wide variety of productive sectors such as food stuffs industry, cosmetics, and medicine. However, the safety of TiO_2_ nanoparticles exposure is still unclear [3]. A considerable amount of literature reports have been published on the toxic effect of TiO_2_ nanoparticles following exposure through different routes (oral, inhalation, and intravenous) [37–39]. Biodistribution studies have reported that titanium nanoparticles accumulate mainly in the liver and kidneys before their excretions, which requires the evaluation of their functional state when the toxicity of titanium has been investigated [39, 40]. In fact, the liver as a vital organ plays a key role in the mechanism of drug metabolism and xenobiotics detoxification; hence, it is highly recommended to evaluate its functional state when the toxic effects of titanium nanoparticles are explored [41]. After the hepatic metabolism of TiO_2_, the kidneys are the principal route of their excretion through glomerular filtration process, and TiO_2_ dioxide nanoparticles accumulate in the kidneys and subsequently induce renotoxicity. A previous study documented that approximately 20% of TiO_2_ nanoparticles persist chronically in the hematopoietic tissue of the kidney and do not reach the kidney tubules to be excreted in the urine [42]. Additionally, due to their small size, TiO_2_ nanoparticles cross the blood–brain barrier and accumulate in the brain, especially in the cortex and hippocampus and thus induce nervous cytotoxicity, inflammation, oxidative stress, and cell apoptosis [3, 43].

In this work and in related references [44, 45], it was observed that nano-TiO_2_ administered orally induced a rise in serum transaminase activities (AST and ALT) as well as plasmatic LDH levels; the most likely explanation of these results is the liver injury due to toxin and, consequently, the leakage of cytoplasmic enzymes [46]. It was found that TiO_2_ nanomaterials induced hepatotoxicity through the over production of reactive oxygen species (ROS) [47]. Most importantly, the administration of bee bread extract alone did not change the plasma levels of these hepatic markers, which proves the safety of the BB extract at the tested dose (100 mg/kg bw). The simultaneous administration of BB extract and TiO_2_ has reduced significantly the plasmatic ALT level and minimize the plasma elevation of LDH and AST levels, which illustrates its hepatoprotective effect. In fact, bee bread is a real source of bioactive molecules with a powerful hepatoprotective effect. Our previous work on the general characterization of the same bee bread sample used in the present study showed its rich composition in phytomolecules such as kaempferol-O-hexosyl-O-rutinosid, quercetin-O-hexosyl-O-hexoside, methylherbacetrin-O-dihexoside, isorhamnetin-O-hexosyl-O-rutinoside, quercetin-O-pentosyl-hexoside, quercetin-3-O-rutinoside, methylherbacetrin-3-O-rutinoside, isorhamnetin-O-pentosyl-hexoside, isorhamnetin-O-rhamnoside-hexoside, and isorhamnetin-3-O-rutinoside. Several studies have been reported the protective effect of quercetin and kaempferol against hepatotoxicity and liver diseases [48–50]. Therefore, the hepatoprotective effect of our sample extract most probably is due to the bioactivity of quercetin and kaempferol as well as the synergistic effect between its particular phytochemical components through different signaling pathways. In addition, the subacute administration of TiO_2_ nanoparticles increased significantly the fasting blood glucose levels (FBGL), which is in agreement with the data of Mao et al. [51]. Recent research has shown that ROS generated following the long term nano-TiO_2_ exposition was found to be the main mechanism associated with high FBGL; in turn, the persistently hyperglycemia triggers the overproduction of ROS and therefore decreases insulin biosynthesis and promotes insulin resistance [52, 53]. The coadministration of BB extract for 30 days (Group 4) was able to reduce hyperglycemia, which is in agreement with the finding of Capcarova et al. [23]. Hypoglycemic activity of BB extract might be due to the interaction between its individual molecules, especially flavonoids vitamins C and B [54]. Indeed, Al-Ishaq et al. reported that flavonoid derivatives reduce hyperglycemia by modulating glucose transport in addition to their powerful antioxidant capacities [55].

Results of the present study reveal a change in lipid profile induced by TiO_2_ nanoparticles; the possible explanation of this result is that shown by Cui and coworkers, which have proven that titanium exposure can disrupt 785 hepatic genes and among those genes responsible for lipid metabolism [56]. In addition, changes in lipid profile following the subacute treatment with TiO_2_ nanoparticles referred to its inhibiting action on lipoprotein lipase activity (a main enzyme involved in the metabolism, transport, and tissue uptake of lipids) [57]. On the other hand, the simultaneous administration of BB extract and TiO_2_ nanomaterials modulates the lipid profile variations, which indicating that BB might reduce the risk and prevent the development of cardiovascular diseases. Similar results were reported by Othman et al. [58]. Growing reports suggest that lipid profile modulation is linked to the activity of caffeic and ferulic acids [59, 60], which are most probably present in the bee bread sample.

As shown in Figure 5, rats treated with TiO_2_ nanoparticles alone (group3) expressed significant elevation of creatinemia, plasmatic urea, and blood electrolytes levels (Na^+^, K^+^, and Cl^−^) accompanied by a decrease in total protein and plasmatic albumin levels. These results go on hand with previous data [30, 44], whereas the concomitant treatment with BB extract showed noteworthy improvement of the major analyzed parameters. In fact, BB is a good source of polyphenols and flavonoids, antioxidants known to be effective against toxicity and renal damage induced by titanium nanomaterials; recently, it was suggested that polyphenols and flavonoids exert their nephroprotective effects following their interaction with the cytoplasmic membrane of renal tubule cells [61].

After reaching the body, TiO_2_ nanoparticles attain tissues through bloodstream. Indeed, the histological examination of the kidney, brain, and liver tissues revealed that TiO_2_ nanoparticles cause serious pathological changes in these tissues. Based on previous studies [45, 62], oxidative stress has been suggested to be the main mechanism involved in TiO_2_ nanoparticle toxicity which can lead to all these observed perturbations of serum biochemical parameters and histological damage of the liver and kidney tissue [63]. TiO_2_ nanoparticle biodistribution studies have shown that they tend to accumulate in the liver and kidneys and they are very difficult to eliminate because of their small size, which leads to damages in the liver and kidneys and causes also a disruption of the biochemical parameters [1].

The issue of neurotoxicity of TiO_2_ nanoparticles has received considerable critical attention; recent studies have shown that TiO_2_ nanoparticles could induce disturbances of brain homeostasis, inflammation, neuron death, and brain injury [63, 64].

Several studies have been conducted to find protective tools against the toxicity of TiO_2_ nanoparticles [45, 62]; to the authors' knowledge, no study has been performed on bee products. The empirical findings of this study showed that the coadministration of bee bread extract and TiO_2_ nanoparticles revealed a potent protective effect against the toxicity of titanium dioxide nanoparticles. The beneficial effects of bee bread could be attributed to its bioactive molecules and antioxidant compounds such as methylherbacetrin, quercetin, and kaempferol derivatives previously detected in the investigated sample [25, 33]; these components are potentially able to protect many tissues against oxidative stress induced by TiO_2_ nanoparticles [65]. Numerous reports have established a link between the beneficial effect of bee bread and its composition in honey and bee pollen which could have a synergistic effect, boosting up its pharmacological activities and its therapeutic properties [17, 18].

## 5. Conclusion

One of the more significant findings to emerge from this study is that bee bread ethanolic extract has a very important protective effect against the majority of metabolic and histological disorders induced by the subacute intoxication of rats with TiO_2_ nanoparticles. Further studies are needed to know the exact protection mechanism and the active ingredient responsible for these beneficial effects.

## Data Availability

The data used to support the findings of this study are included within the article.
